# Performing Dual Glucose Clamp Experiments in Sedated Farm Swine: A Practical Method

**DOI:** 10.3390/mps8050118

**Published:** 2025-10-02

**Authors:** Marc C. Torjman, Winston C. Hamilton, Katherine Dillon, Channy Loeum, Jeffrey I. Joseph

**Affiliations:** Department of Anesthesiology and Perioperative Medicine, Sidney Kimmel Medical College, Thomas Jefferson University, Philadelphia, PA 19107, USA; marc.torjman@jefferson.edu (M.C.T.); kate.dill94@gmail.com (K.D.);

**Keywords:** glucose clamp, hyperinsulinemic, euglycemic, infusaport, swine, sedation, anesthesia

## Abstract

The hyperinsulinemic–euglycemic clamp technique is considered the gold standard for measuring insulin sensitivity in large animals. We developed a practical method for conducting concurrent glucose clamp experiments in a pair of sedated farm swine positioned in a sling. Descriptions of customized equipment and central venous access surgical procedures for blood collection are provided. Personnel functions are described for execution of the clamp protocol. A total of 24 hyperinsulinemic–euglycemic clamp studies were performed over 6 weeks. Infusaports remained functional for 1454 blood samples. There were three CSII catheter occlusions during bolus administration, and the swine showed no signs of infection or disease. IM telazol at 1.0 mg/kg, administered 1–2 h prior (mean of 3.26 mL ± 1.59) was effective in keeping animals comfortable. SpO_2_ and heart rate remained within normal ranges. Means ± SD total infused volumes for octreotide, 10% dextrose, and saline were 9.7 ± 0.93 mL, 2328.0 ± 672.8 mL, and 690.3 ± 206.8 mL. Mean blood glucose was maintained between 75.7 and 87.8 mg/dL (CV 3.17%) for the 24 experiments. The GIR infusion rate peaked between 15 and 60 min after insulin bolusing, with insulin C_max_ of 108.5 pmol/L and t_max_ at 10 min. All aspects of the protocol were effectively carried out. The animals remained in good health, and the implanted infusion ports remained patent for over 700 blood draws per animal. This method could potentially reduce the number of animals used and the costs of other similar experiments.

## 1. Introduction

Pre-clinical studies using clamp techniques in large animals are essential for assessing insulin pharmacokinetics and pharmacodynamics (PK/PD) [1,2], glucose metabolism [3], and for testing new glucose biosensors [4]. Although non-human primates are ideal animal models for such studies, due to their genetic and physiological proximity to humans, those investigations are less frequently planned because of ethical and high-cost considerations [5,6]. Canines have a long history in diabetes research [7,8]; however, their low amount of subcutaneous tissue is a limitation to modeling human subcutaneous insulin absorption kinetics. This feature also does not permit obtaining a sizable tissue specimen, which is important for histology and three-dimensional imaging studies to examine insulin travel through the extracellular matrix. The most complete studies investigating continuous subcutaneous insulin delivery and glucose control require examination and correlation of histological findings, an important advantage of the swine model [9,10]. Swine have also been a preferred model due to their anatomic and physiologic similarity to humans [11,12,13].

The hyperinsulinemic–euglycemic clamp technique [14] is still considered the gold standard for measuring insulin sensitivity to subcutaneously administered insulin. The protocols are labor-intensive, often requiring studies over days or weeks, with frequent blood sampling from the central circulation. With our extensive work in this area, this laboratory has developed a practical method for conducting concurrent glucose clamp experiments in two sedated farm swine each positioned in a sling for a 4 h study. This specific methodology could benefit researchers in this field, or other areas of research where non-ambulatory sedated swine may be used. Of importance is that this method accommodates study designs for repeated survival experiments in the same animals, potentially reducing the number of required animals in similar studies. This manuscript describes an efficient and effective method for performing hyperinsulinemic–euglycemic clamp experiments in sedated swine pairs.

## 2. Experimental Design

Approval for the use of animals was first obtained from the Thomas Jefferson University Animal Care and Use Committee. The study protocol was designed to evaluate the PK/PD responses to continuous subcutaneous insulin infusion (CSII) using dosing equivalent to a pre-meal bolus in a type 1 human diabetic. For successful frequent blood sampling and repeated infusions, central venous access was necessary. Surgical implantation of two blood sampling ports and placement of insulin delivery catheters under general anesthesia was performed prior to initiation of the study.

### 2.1. Animal Handling and Apparatus

**Figure 1:**
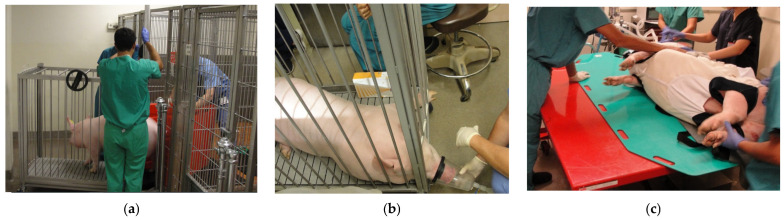
Panel (**a**): Illustration of the use of a bi-fold panel to move a swine into the transport cage. The animal handler used the red bi-fold panel opened in a V shape to move the animal forward while preventing it from turning around. Panel (**b**): Induction of anesthesia using isoflurane administered via a nose cone prior to moving the animal onto the hydraulic lift table. Panel (**c**): Animal moved from the hydraulic lift table to the operating table.

Two five-month-old female farm swine were obtained from Animal Biotech Industries (Danboro, PA, USA) two weeks prior to the start of the study. After acclimation to their new environment, the animals were fasted for 12 h, as the study procedures and experiments required administration of sedatives and general anesthesia. In order to safely move these large animals to the procedure area, the swine were coaxed into a transport cage using a bi-fold panel (Figure 1a). They were then sedated with 1–2 mg/kg Telazol (Zoetis, Kalamazoo, MI, USA, stock supplied as 100 mg/mL), administered via intramuscular injection (IM) in the buttocks [15]. The transport cage was wheeled to the procedure room, and the team waited for the sedation to take effect, typically within 10–15 min. This was evident when the swine became sleepy and laid down in the transport cage. Oxygen (100%) and 0.5–2% isoflurane were administered via a large nose cone placed under the partially raised cage gate (Figure 1b). An intramuscular dose of atropine (0.01–0.02 mg/kg) was given at this time. Once fully sedated, the front and back gates of the transport cage were removed, with two team members positioned at either end of the transport cage. A tarp with handles was slid under the animal, and in a coordinated effort, the first swine was moved from the transport cage to the floor.

At this point, swine positioning and anesthesia varied according to one of the three procedures required for the experiment: (a) implantation of sampling port catheters, (b) CSII catheter placement, and (c) PK/PD clamp studies. Procedures a and b (venous port and CSII catheter placement) each required general inhalation anesthesia prior to moving the swine from the floor onto the operating table using a hydraulic lift (Figure 1c).

For the PK/PD clamp studies (c), the same anesthetic technique was used to anesthetize the animal in the transport cage and place the animal on the floor in the supine position. Two team members then placed a large animal canvas sling (Lomir Biomedical, Inc.; Notre-Dame-de-l’Île-Perrot, QC, Canada/Malone, NY, USA) over the legs of the supine swine to position the animal in a customized support frame designed and built by the investigator. A description with illustrations is provided in Section 2.5.

**Figure 2:**
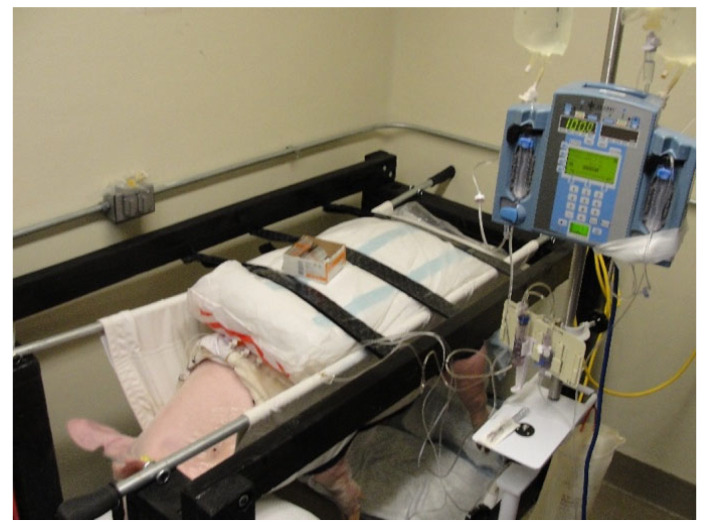
An animal is shown supported within the sling, with its 4 limbs hanging freely. The steel rods passed through the sling were used to lift the animal for positioning into the frame. The steel rods were secured between heavy-duty eye bolts at the 4 corners of the frame. Three Velcro straps across the swine’s back served as restraints to prevent upward movement.

Two memory foam cushions were inserted between the sling and the swine to prevent the leg holes from causing pressure and discomfort around the swine’s shoulders and hips. The anterior cushion also allowed the swine to rest its head on the front part of the sling, with the four legs approximately six inches from the floor. Dorsal cushions secured with Velcro^®^ brand straps prevented the swine from potential bucking during the experiments (Figure 2). More details of these three procedures are provided in the other sections below.

### 2.2. Anesthetic Technique for Catheter Ports and CSII Placements

The swine were sedated one hour apart with 1–2 mg/kg telazol IM for transport to the operating room. The operating room was set up with the standard anesthesia monitors consisting of a Midmark anesthesia workstation (Midmark Corporation, Versailles, OH, USA) capnography, pulse oximetry, a rectal temperature probe, and inhalation agent and oxygen gas analyzers. General anesthesia was induced with isoflurane using 1.0–1.5 MAC, and atropine (0.01–0.02 mg/kg IM) was administered as indicated to maintain a normal heart rate. For catheter port placement, endotracheal intubation was performed and mechanical ventilation was initiated using a Midmark ventilator (model 9180081, Midmark Corporation, Versailles, OH, USA). The tidal volume was set at 10 mL/kg and the respiratory rate at 18–25 breaths per minute, with adjustments based on P_ET_C_O2_. Arterial oxygen saturation was maintained at 100% with an FI_O2_ of 90–100%. For subcutaneous catheter placement, mask ventilation was used to deliver isoflurane, and atropine was administered at induction as previously indicated. For both procedures, body temperature was maintained at 37 ± 1 °C using three heating pads placed under the swine for the duration of the procedure.

### 2.3. Implantation of Central Venous Catheter Ports

**Figure 3:**
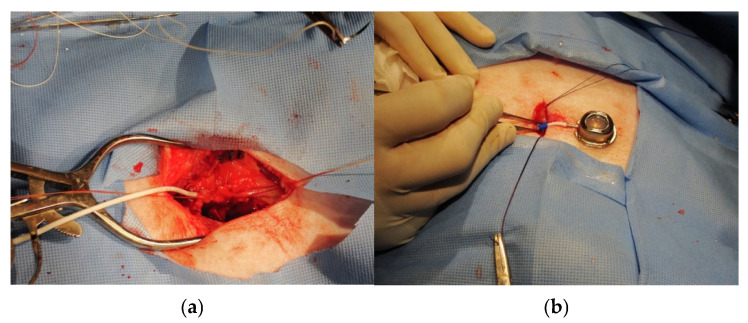
Panel (**a**): Isolation of the internal jugular vein with inserted central venous catheter (white). The catheter was a 7 French rounded tip silicone central venous catheter, 60 cm in length. Panel (**b**): Accessible vascular port ready for insertion into subcutaneous pocket on the superior lateral region of the animal’s neck.

There are several considerations in this procedure that are specific to swine: (1) Positioning the port in the superior lateral region of the animal’s neck prevents the animal from reaching their incision sites and protects those sites from rubbing against the pen bars. (2) Placement of the vascular access port with cervical incisions caudal to the ears allows for easy access to the jugular veins. (3) Isolation of the internal jugular vein requires careful identification and good surgical skills for separation, but it is possible in swine given their anatomy [16,17,18]. We selected a central venous catheter (CVC) with an accessible vascular port (Swirl Max attachable 7FR rounded tip Silicone Catheter, 60 cm in length, Access Technologies, Skokie, IL, USA) for insertion into the left and right internal jugular veins (Figure 3a). Following catheter insertion, the accessible port was placed in a created subcutaneous pocket on the superior lateral region of the animal’s neck (Figure 3b). For this procedure, the swine were placed in the dorsal Trendelenberg position on the operating table, pulling the forelegs caudally. The incision site was then washed with soap and sterile water, shaved with coarse and fine clippers, and the skin was prepped with Chlorhexidine. A one-time intravenous dose of 17 mg/kg Cephazolin antibiotic (1 g/10 mL vial, Westward pharmaceuticals) was administered prior to skin incision to reduce the risk of infection. A sterile irrigation bowl was also filled with 250 mL of saline and 10 mL of Cephazolin for wound irrigation. The procedure was conducted in two steps using the standard sterile technique for surgery. First, a skin incision of approximately 7 cm was made on the ventral neck above and in the same direction as the internal jugular vein. The vein was exposed using blunt dissection by carefully spreading the tissues surrounding the vessel. Once the internal jugular vein was sufficiently exposed to permit visualization of at least 5 mm of the vessel diameter, the catheter was inserted into the internal jugular vein and advanced 3–4 cm into the vessel. The catheter was secured by tying two previously placed loose sutures around the catheter and vessel. Using a trocar, the catheter was then tunneled to the dorsal neck caudal to the ears where a second incision was made for the creation of a subcutaneous pocket, where the port was placed. The wound was irrigated with antibiotic solution and closed (two layers) using numbers 2, 3, and 4 vicryl sutures and Steri-Strips^TM^ (3M Center, St. Paul, MN, USA).

After covering the incision with a Tegaderm clear dressing (Tegaderm^TM^ (3M Center, St. Paul, MN, USA), the procedure was repeated on the opposite side. The swine were administered intramuscular buprenorphine (0.05 mg/kg) approximately 60 min prior to emergence from general anesthesia and monitored for signs of infection for 48 h. Postoperative recovery lasted 14 days, which was sufficient for the closure of incisions. The animals were monitored by veterinary staff and examined daily by the institutional veterinarian to ensure that the swine had no post-surgical complications and were in good health. During this recovery period, both catheter ports were flushed initially with 10 mL of normal saline, followed by 1.5–1.8 mL Taurolidine-Citrate lock solution (TCS-04TM, Skokie, IL, USA), and every 3–4 days for maintenance as per manufacturer’s instructions. The catheters were also flushed at the conclusion of every glucose clamp experiment. Our goal was to maintain catheter patency for one month, although successful use of these devices for blood draws has been demonstrated from months to years [17].

### 2.4. Insertion of Continuous Subcutaneous Insulin Delivery Catheters

**Figure 4:**
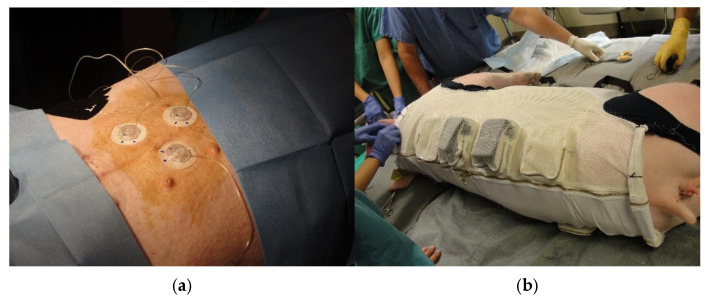
Panel (**a**): Inserted commercial subcutaneous insulin delivery catheters connected to portable insulin pumps. The catheters were then protected with Tegaderm dressing and covered with stretchable KT tape prior to fitting of the swine’s vest. Panel (**b**): Placement of the animal’s vest while under light isoflurane anesthesia. Insulin pumps were secured in foam padded zippered pockets.

Commercial CSII catheters (Medtronic MiniMed Quick-Set, 6 mm catheter length, 90 degree insertion) were inserted into the subcutaneous tissue of the abdomen (Figure 4a) under general inhalation anesthesia with spontaneous ventilation. These catheters are FDA-approved for human use, and each set of catheters was used for 4 PK/PD studies. Pre-operatively, the swine were sedated with 1–2 mg/kg telazol and placed on the operating room table in the supine position using masked isoflurane general anesthesia. The insertion sites were prepped as described above. The insulin delivery catheters were inserted using commercial applicators, and each catheter was covered with a piece of gauze cut to fit over the catheter housing, followed by application of Tegaderm^TM^ clear dressing and therapeutic kinesiology tape. The gauze over the catheter body permitted easy removal of the adhesive and good visual inspection of the catheter before and during removal. A thin stockinette was then slid over the animal, with 4 holes cut out for the legs. This stretchable garment added further protection to the insulin delivery catheters. The swine were then fitted with a Lomir vest designed with padded pockets to hold the insulin pumps attached to the catheters. Thus, the catheters were protected by Tegaderm dressings, kinesiology tape, and a stockinette. Insulin pump protection in the vest pockets was provided by inserting the pumps into customized foam pads within the pockets (Figure 4b).

One CSII was initiated with commercial insulin lispro (U-100, Eli Lilly and Company, Indianapolis, IN, USA) at 0.4 units/hour, and one with preservative-free saline or diluent via 2 commercial insulin pumps. This strategy was used to mimic real-world conditions and avoid potential occlusion of the catheters, while comparing occlusion rates between insulin and saline infusions. Postoperatively, the swine were monitored twice daily for signs of pain/discomfort, hypoglycemia, and infection. Glucose concentrations (trends) were also monitored using two real-time continuous glucose sensors (CGM G4 Dexcom Inc, San Diego, CA, USA) placed laterally on the lower abdomen.

### 2.5. Canvas Sling and Support Frame for PK/PD Experiments

**Figure 5:**
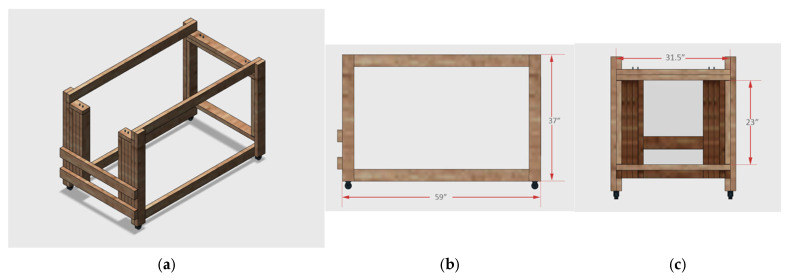
Diagram of the customized support frame designed to hold the animal in a sling for the glucose clamp experiments. The frame was constructed using 2 × 4 higher-quality lumber that was painted and seal-coated prior to use. Panels (**a**–**c**) show a top view and side views of the width and height, respectively.

PK/PD studies were performed with the swine supported in a canvas sling that was suspended by two galvanized steel rods secured within a wooden support frame (Figure 2 and Figure 5). Multiple commercial frame models were considered for the frame, but it proved to be more practical to construct a custom frame that could easily accommodate 40–90 kg swine. A final wood frame design was reviewed and approved by the laboratory team and animal facility veterinarian prior to construction. As illustrated in the schematic drawing, the wooden framework was assembled using 2 × 4 lumber that was painted and seal-coated. The two frames were outfitted with two sets of four-inch wheels, three industrial-strength Velcro^®^ brand straps for securing the swine from above, and eight zinc-plated eye bolt screws to hold the sling rods. The steel rods were fixed between zinc-plated eye bolt screws. The two support frames constructed allowed the animals to remain in a non-weight bearing position for the duration of each PK/PD study.

**Figure 6:**
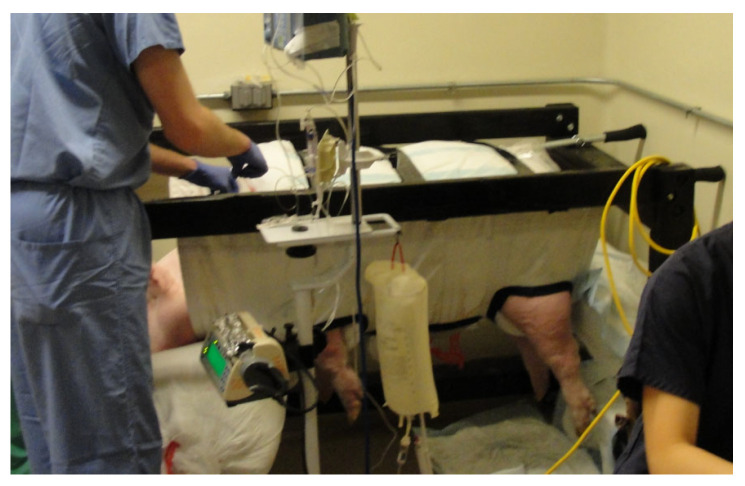
Sedated swine positioned in the sling with foam padding to support the head and belly of the animal.

The rods were threaded through two sleeves sewn along the entire length of both sides of the canvas sling, supporting the weight of the suspended swine (Figure 2). For additional grip, rubber bicycle handles were inserted over both ends of the steel rods. It was important that the rods could be rapidly inserted into the sling after it was fitted over the abdomen and chest through the 4 leg holes on the sedated supine animal. The animal was then rotated onto its flank and then lifted for placement within the frame by four individuals, each holding one end of a steel rod. This procedure was reversed at the end of the clamp study, and the two rods were removed before moving the animal from the ground and into the transport cage. The canvas sling is washable and designed for large animal research. It supports the swine from below and has a rectangular pocket cut out of the canvas panel that allows the abdomen to hang freely. This maximizes animal comfort while minimizing abdominal blood flow obstruction. (Figure 6). The latter was important for this experiment, as subcutaneous insulin delivery catheters were inserted in the subcutaneous tissue of the swine’s abdomen.

### 2.6. Hyperinsulinemic–Euglycemic Clamp

(a) First animal

The clamp protocol described is specifically designed to study the PK/PD properties of subcutaneous insulin administration in swine; however, it may also be used for intravenous insulin administration studies. The only modification would be the omission of CSII catheter placement and the insertion of an additional dedicated intravenous catheter for infusion of insulin.

**Figure 7:**
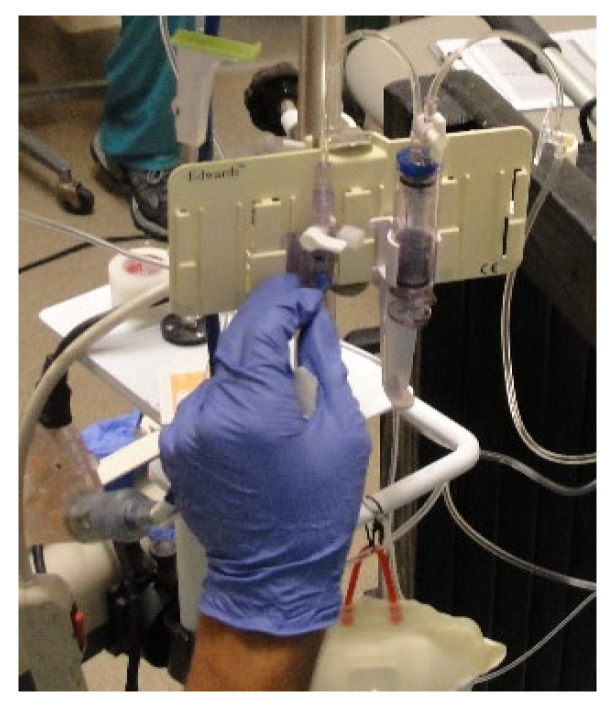
Needleless VAMP Plus blood sampling system attached to one of the two infusaports for closed blood sampling through a z-site self-sealing sample port. Pressurized 1 L 0.9% NaCl solution was used for flushing by pulling on the elastic pigtail.

The preparation of supplies and equipment for each euglycemic clamp study took place approximately one hour before the first swine was transported to the procedure room. Once the first swine was sedated and situated in the sling and frame, Chlorhexidine swabs were used to clean the infusaport sites. Wearing sterile surgical gloves, a right-angle Huber needle was inserted into the infusaport, and up to 5.0 mL of blood was withdrawn to verify catheter patency. The line was subsequently flushed with 10 mL 0.9% NaCl solution, and the right infusaport was attached to three stopcocks placed in series. Attached to the first stopcock was the octreotide infusion, the second stopcock was for normal saline, and the third was attached to the 10% dextrose (D10) infusion to control the Glucose Infusion Rate (GIR) throughout the experiment. The second stopcock with normal saline solution was used to maintain a constant infusion of octreotide. The left infusaport was attached to a VAMP Plus system (VP2 blood sampling system, VMP400 Needless Cannula, PX284 pressure transducer, Edwards Lifesciences Corporation, Irvine, CA, USA) for closed blood sampling through a z-site self-sealing sample port. Attached to the VAMP Plus system was a 1.0 L 0.9% NaCl solution for flushing (Figure 7).

After the infusaports were properly accessed and the lines set up correctly, the four team members required to run the study assumed their positions (see Appendix A for study team member responsibilities).

Once the fasting animal was comfortably positioned in the sling apparatus, endogenous insulin secretion was suppressed using an octreotide infusion. An initial octreotide bolus dose (7.2 µg/kg) was administered 60 min prior to starting the clamp, followed by a maintenance dose of (3.6 µg/kg/h). During this first hour, 10% dextrose solution (D10) was titrated to gradually raise the animal’s blood glucose to 85 mg/dL, being careful not to overshoot this target concentration. The clamp procedure began upon bolus insulin administration (0.15 IU/kg), followed by D10 infusion to maintain glucose concentrations at 85 mg/dL (±10%). Blood samples were drawn at 5 and 10 min intervals for glucose and insulin, and GIR infusion was adjusted accordingly.

(b) Second animal

**Figure 8:**
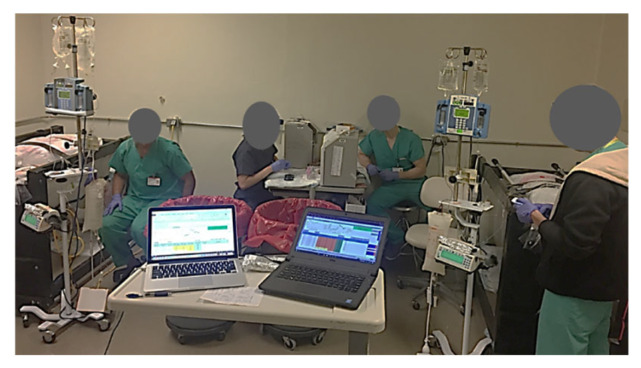
Illustration of the experimental setup with the two swine in their respective slings. Four of the team members are shown in their respective positions: 2 YSI (YSI Inc., Yellow Springs, OH, USA) operators for blood glucose analysis and 2 VAMP (Venous Arterial Blood Management Protection) operators for blood draws. Not shown are the GIR monitor/swine soother and the glucose clamp operator.

After beginning the clamp study on the first swine, a second team ensured that duplicate laboratory and anesthesia equipment were set up and calibrated in the other half of the procedure room. The same transport and preparation procedures were performed for swine #2, and the clamp procedure was initiated with an approximate 45 min offset in start time between the two swine, and 2.5 min between the sampling intervals. Due to the relatively short duration of action of telazol in swine, additional bolus doses (1–2 mg/kg IM) were administered as necessary, with careful monitoring of respiration and oxygenation. The final dose was given approximately 30 min prior to the end of the study for transport back to the animal care facility. Data were therefore collected simultaneously in the two clamped animals using duplicate equipment for the study protocol (Figure 8). The study data included time of blood draw, glucose YSI and meter concentrations, GIR, time of GIR change, drug and fluid administration, vital signs, and comments. Blood glucose measurements and insulin samples were obtained at 5 and 10 min intervals for 240 min, while other data, such as vital signs and gas concentrations, were recorded continuously.

Data were recorded using Microsoft Excel Version 2016. Descriptive statistics are provided as means ± SD using IBM SPSS Statistics Version 24.0 and GraphPad Prism Version 9.0.

## 3. Results

The entire 10-week study period included the acclimatization of the animals (2 weeks), followed by access port implantation and the recovery period (2 weeks). The swine had no signs of infection or disease from natural or iatrogenic causes throughout the 10-week study period. A total of 24 glucose clamp experiments were performed using the two constructed support frames over a 6-week period. The use of 1.0 mg of IM telazol every 1–2 h (mean of 3.26 mL ± 1.59) was effective in keeping the animals calm and comfortable, in addition to the initial doses of telazol used in transport. SpO_2_ and heart rate were monitored and remained within normal ranges: 95–100% and 60–125 bpm, respectively. There were three CSII catheter occlusions during bolus administration at the start of clamps 3, 4, and 10 in the same animal, most likely related to kinking of the catheters. The infusaports were easily accessed and remained functional for the duration of the study, with a total of 1454 blood samples collected from the two animals. A total of four samples were missed due to difficulty with those draws within the five-minute sampling interval of the protocol.

**Figure 9:**
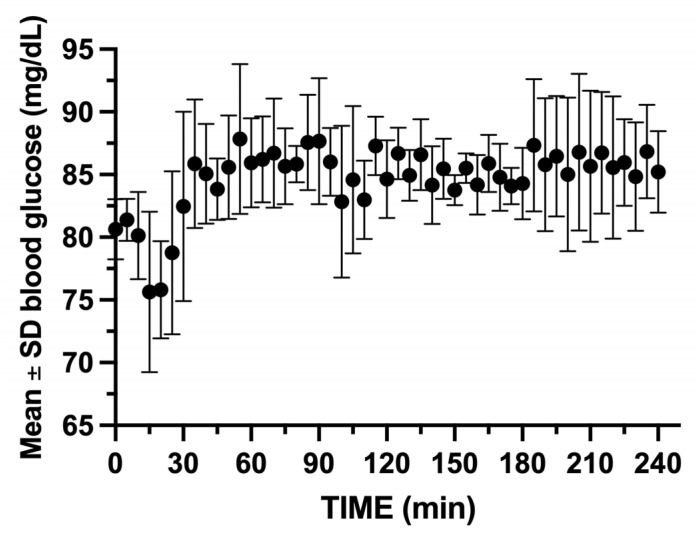
Mean ± SD blood glucose for the 2 swine during the 24 glucose clamp studies. Note the initial drop in glucose after insulin bolus administration. GIR must be rapidly increased to maintain euglycemia.

During the entire study period, the two swine’s weights increased from a mean of 46.13 ± 0.32 kg upon arrival, to 67.00 ± 1.41 at the start of the glucose clamp experiments. Their final mean weight prior to glucose clamp #24 was 81.00 ± 5.65 kg. The mean ± SD total infused volumes for octreotide, 10% dextrose, and saline were 9.7 ± 0.93 mL, 2328.0 ± 672.8 mL, and 690.3 ± 206.8 mL, respectively. The mean blood glucose was maintained between 75.7 and 87.8 mg/dL (CV 3.17%) for the 24 experiments (Figure 9).

**Figure 10:**
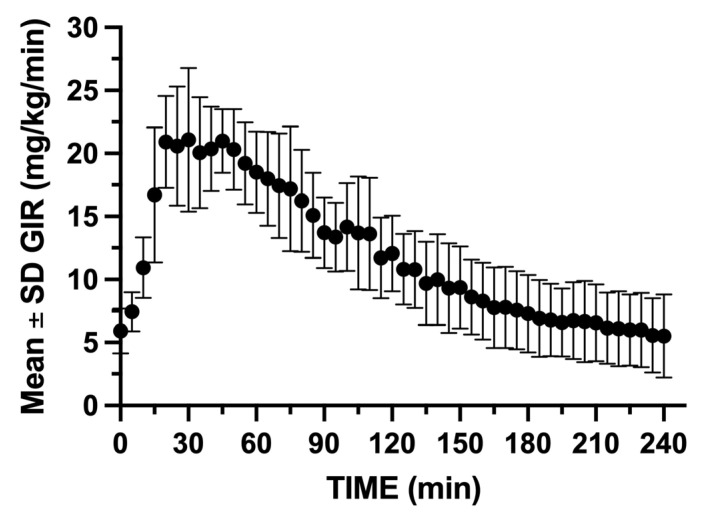
Mean ± SD GIR during the 240 min hyperinsulenemic euglycemic clamp. The glucose clamp operator needed to remain vigilant to the glucose concentrations following insulin bolus and was required to rapidly increase GIR to maintain the target glucose range.

**Figure 11:**
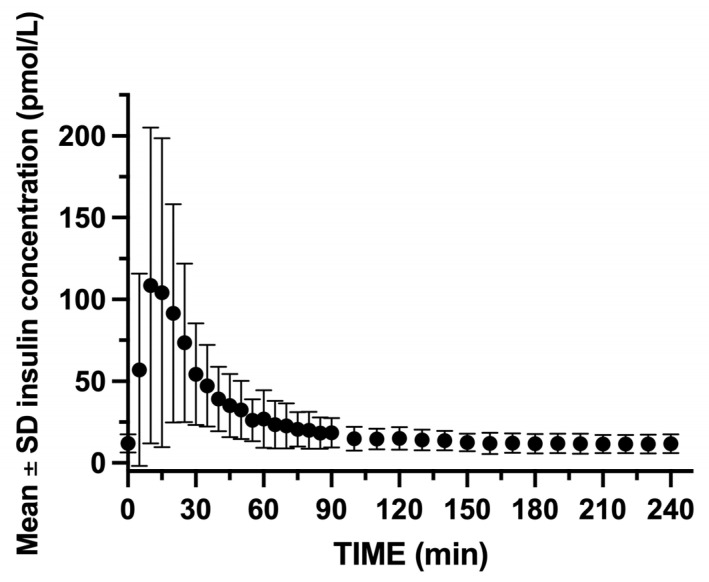
Mean ± SD insulin concentrations for the 2 swine during the 24 glucose clamp studies. The insulin concentration peaks within 15 min after insulin bolus administration.

GIR was rapidly increased within the first 5 min following insulin bolus administration, reaching a peak infusion rate between 15 and 60 min following insulin blousing (Figure 10). Insulin concentrations peaked rapidly with insulin C_max_, reaching 108.5 pmol/L, and t_max_ occurred at 10 min post-bolus. The insulin concentrations gradually decreased, returning to near baseline by 90 min (Figure 11).

## 4. Discussion

The hyperinsulinemic–euglycemic clamp technique has traditionally been useful to study the PK/PD of insulin formulations [19], but has recently become a valuable technique in the fields of neuro-engineering and bioelectronic medicine [20,21]. Based on the fact that the swine are required to have up to 7 days of recovery after each glucose clamp, some investigators may find our approach to simultaneously study swine pairs useful. Additionally, as these experiments are typically repeated multiple times in the same animal, this approach could save time and cost by reducing the total effort necessary to perform these complex studies.

We attribute the success and effectiveness of this method to three key factors: (1) selection of the proper equipment suitable for these experiments, (2) recruitment of a team of individuals cross-trained to perform the required tasks, and (3) appropriate administration of anesthesia and good surgical and animal handling skills. The members of the team were selected several weeks prior to the start of the experiments, and the two swine were delivered to the vivarium two weeks prior to the start of the study. This approach facilitated early planning and implementation of the mandatory staff training required for the 24 glucose clamps. Although the time commitment from the faculty investigators was high, this was necessary to ensure appropriate education and also allowed for input from team members. Additionally, when difficulty was encountered at a station, the fact that team members were cross-trained to perform all study-related functions allowed for continuity of protocol execution. We believe that the combination of staff training and proficiency in surgery and anesthesia contributed to the efficiency and safe conduct of these experiments.

An important recommendation for reproducibility in future work is to remain proactive with sedating the swine during the PK/PD studies. The administration of sedation should occur before the animal becomes overly agitated, which may also make it more difficult to predict and adjust GIR to maintain euglycemia.

The animal’s position in the canvas sling should be monitored, and head cushions should be placed between the swine’s front and back legs and adjusted periodically for comfort and support. The anterior cushion prevents the swine’s head from hanging down, which could compromise blood flow to the SVC and result in difficulty with blood draws. The latter could be quickly resolved by leveling the head and moving it to the contralateral side of the CVC port being used for the draws.

Finally, over the course of the study period, the swine’s weights increased from approximately 65 to 85 kg. The canvas slings were replaced with larger slings when indicated, and similarly, the animals’ vests were adjusted by adding dorsal expansion panels with dual zippers. The use of a smaller sling than required could increase agitation and the need for more sedation. We would therefore recommend having a larger sling available for the latter period of the study.

## 5. Conclusions

Although this protocol would be considered to have a relatively high degree of difficulty, all aspects of the study were effectively carried out by a team of eight cross-trained individuals assigned to workstations with specific functions. Customization of some of the equipment, combined with well-planned study procedures, provided a system that contributed to the flow and success of the study. The animals remained in good health for the duration of the study, with no infection or distress, and the implanted vena cava infusion ports remained patent long-term, allowing for over 700 blood draws per animal. We believe that the ability to effectively and safely use the same animals for multiple experiments could potentially reduce the number of animals and costs to support other similar experiments.

## Data Availability

This paper focuses on detailing the unusual and practical methodology used for the experiments. Data sharing is not applicable to this article.

## Abbreviations

The following abbreviations are used in this manuscript:

IM	Intramuscular
SpO_2_	Oxygen saturation by pulse oximetry
PK/PD	Pharmacokinetics and pharmacodynamics
CSII	Continuous subcutaneous insulin infusion
P_ET_C_O2_	Partial pressure of end tidal CO_2_
FI_O2_	Fraction of inspired oxygen
CVC	Central venous catheter
VAMP	Venous arterial blood management protection
GIR	Glucose infusion rate
YSI	Yellow Springs Instruments blood glucose analyzer

## Appendix A

### Study Team Individual Responsibilities

(1) VAMP Operator (Blood Draws): This individual was trained to use the VAMP Plus System for closed blood sampling every 5 min, and they informed the Glucose Clamp Operator of the draw time. After handing over the blood sample to the YSI Operator for glucose analysis, this individual was responsible for flushing the sample line and the administration of additional fluid when required. One VAMP Operator was assigned per swine.
(2) Laboratory Technician (YSI Operator and sample processing): This individual was trained to operate the YSI 2300 glucose analyzer, the point-of-care glucose monitors, and to process and store samples for later assays. Upon receipt of the blood sample from the VAMP Operator, he/she introduced the blood into three Abbott Freestyle Lite glucose monitors and recorded those values. The operator then transferred blood aliquots into designated vials and presented the remaining samples to the YSI 2300 STAT Glucose Lactate analyzer (YSI Inc., Yellow Springs, OH, USA). The results were immediately called out to the Glucose Clamp Algorithm Operator. One Laboratory Technician was assigned per swine.
(3) Glucose Clamp Operator: The operator was positioned between the two animals with two laptops (one for each swine) on a rolling table and in direct view of the glucose, saline, and octreotide infusion pumps. This position required vigilant monitoring of the blood glucose values, with good judgement of expected BG changes after insulin administration. Experience is important for the forward-looking assessment of BG, which allows for proper gauging of the glucose infusion rate (GIR) at five-minute intervals to maintain the BG in a pre-selected range. The protocol criteria in this laboratory was to maintain a BG level of 85 mg/dL ± 10%. Immediately upon receipt of the YSI BG level, the operator called out the new GIR and recorded the BG, GIR, and times. One Glucose Clamp Operator was assigned per two swine (two rotating supervising faculty).
(4) GIR Monitor and Swine Soother: This was a two-function position, where the individual was positioned by the animal’s head and trained to keep the animal calm and comfortable by providing sedation (telazol with IM injection), petting, and adjusting the foam pads under the animal as required. Their second function was to perform the GIR pump adjustments given by the Glucose Algorithm Operator every five minutes, as well as to replace empty infusion bags. This individual should be familiar with the infusion pumps and be able to rapidly replace fluid bags, as well as troubleshoot and correct pump malfunctions. There are instances when this person is required to assist the VAMP Operator in repositioning the animal’s head and neck during difficult blood draws due to positional occlusion of the vein. One GIR Monitor and Swine Soother was assigned per swine.
